# Genome-wide identification and characterisation of Toll-like receptors in Chinese spiny frog (*Quasipaa spinosa*)

**DOI:** 10.3389/fgene.2025.1569669

**Published:** 2025-06-06

**Authors:** Zehong Li, Zihan Gao, Dexin Mo, Zifeng Zhu, Jingqi Zhang, Mujin Liu, Han Xiao, Meng Zhou, Teng Gao, Rishen Liang

**Affiliations:** ^1^ Innovative Institute of Animal Healthy breeding, College of Animal Sciences and Technology, Zhongkai University of Agriculture and Engineering, Guangzhou, China; ^2^ Yingde Yingxin Agriculture Co., Ltd., Qingyuan, Guangdong, China; ^3^ Laboratory of Animal Disease Detection, Qingyuan Animal Disease Prevention Control Center, Qingyuan, Guangdong, China

**Keywords:** *Quasipaa spinosa*, genome-wide identification, Toll-like receptor, Elizabethkingia miricola, immune response

## Abstract

**Introduction:**

Toll-like receptors (*TLR*s) are pattern recognition receptors essential for immune defense against pathogens, activating the host’s immune response by recognizing conserved pathogen structures. The Chinese spiny frog (*Quasipaa spinosa*), an amphibian native to southern China and northern Vietnam, has been severely impacted by recent infectious disease outbreaks caused by bacterial, viral, and parasitic infections, which threaten the sustainable development of the *Q. spinosa* farming industry. However, the roles of *Q. spinosa* *TLRs* (*QsTLRs*) in combating these exogenous pathogens have not yet been explored.

**Methods:**

In the study, using the whole genome data of *Q. spinosa*, bioinformatics tools were employed to identify and analyze the *TLR* gene family. The bacteria *Elizabethkingia miricola*, a common pathogen, which causes the cataract disease and can lead to serious death of the frog. Here, we selected the bacteria to conduct the challenge experiment in order to characterize the immune responses of the *TLR* genes of *Q. spinosa* against bacterial infection.

**Results:**

The analysis identified 17 members of the *TLR* gene family in *Q. spinosa*. Phylogenetic analysis revealed that *QsTLRs* can be classified into seven subfamilies: *TLR1*, *TLR3*, *TLR4*, *TLR5*, *TLR7*, *TLR11*, and *TLR13*. Conserved synteny analysis indicated that *Q. spinosa* is more closely related to *Rana temporaria* than to *Xenopus laevis*. Protein structure prediction and motif analysis demonstrated that all *QsTLRs* are relatively conserved in both structure and function. mRNA expression levels of *QsTLRs* in spleen tissues were measured following stimulation with *Elizabethkingia miricola*, which revealing that 15 *QsTLR* genes exhibited up-regulation at various time points post-stimulation.

**Discussion:**

These findings provide a comprehensive understanding of the *QsTLR* gene family and lay the groundwork for future studies exploring the functional evolution of the amphibian *TLR* gene family.

## 1 Introduction

Toll-like receptors (*TLR*s) are pattern recognition receptors that detect conserved pathogen-associated molecular patterns (PAMPs), playing a critical role in immune defense against pathogen invasion (Akira et al., 2006). As transmembrane (TM) proteins, *TLR*s initiate the production of immune effector molecules by recognizing conserved pathogen structures. Each member of this extensive family has the function of specifically distinguishes between pathogen classes, coordinating appropriate adaptive immune responses (Takeda and Akira, 2015). *TLR*s are defined as type I transmembrane receptors characterized by leucine-rich repeats (LRRs) in their extracellular domain and a Toll/interleukin-1 (IL-1) receptor domain (TIR domain) in the C-terminal (Mills et al., 2000). Upon LRR recognition of a specific pathogen ligand, the TIR domain recruits downstream signaling proteins, forming a cascade that promotes the production of pro-inflammatory cytokines and interferons (IFNs), ultimately aiding in pathogen clearance (Lim and Staudt, 2013). Through different *TLR*s, the body recognizes potential pathogens and activates the immune response. *TLR3*, *TLR7*, *TLR8*, *TLR9*, *TLR10*, *TLR11*, *TLR12*, and *TLR13* are localized in endosomal membranes, where they detect nucleic acids or proteins. Specifically, *TLR3* recognizes double-stranded RNA (dsRNA) and mRNA, *TLR7* detects single-stranded RNA (ssRNA), immunoadjuvants, and guanosine, *TLR8* responds to immunoadjuvants, ssRNA, and uridine, *TLR9* identifies single-stranded unmethylated 5′-C-phosphate-G-3′ (CpG)-DNA and 5′-xCx DNA sequences, *TLR10* recognizes dsRNA, *TLR11*/*12* complex detects profilin, and *TLR13* targets bacterial 23S ribosomal RNA (Alexopoulou et al., 2001; Jurk et al., 2002; Diebold et al., 2004; Hidmark et al., 2012; Raetz et al., 2013; Lee et al., 2018; Ohto et al., 2018). Previous studies have identified 13 *TLR* members in mammals, each functioning as sensors for distinct PAMPs. A total of 27 *TLR* family members have been identified in vertebrates (Zhang et al., 2017), with 10 members *(TLR1-TLR10*) found in humans (*Homo sapiens*) and 13 members (*TLR1*-*TLR9*, *TLR11*-*TLR13*) in mice (*Mus musculus*) (Holcombe et al., 2011). At least 21 *TLR*s have been identified in teleosts, including several “non-mammalian” *TLRs* such as *TLR18*-*26*. In amphibians, *TLR* family investigations have been reported for *Xenopus* (*Xenopus laevis*) and two salamander species (*Lissotriton montandoni* and *Lissotriton vulgaris*), with 19 *TLR* genes identified in *Xenopus* (Ishii et al., 2007) and 16 in salamanders (Babik et al., 2014). These species also possess non-mammalian *TLRs* such as *TLR19*, *TLR21*, and *TLR22*, although their numbers are fewer than those found in fish. Amphibians, living both in aquatic and terrestrial environments, may have evolved a unique *TLR* family to adapt to their complex habitats (Ishii et al., 2007). To test this hypothesis, further studies on a broader range of amphibian species are needed to investigate their *TLR* gene families.

The Chinese spiny frog (*Quasipaa spinosa*), also known as the stone frog or rock frog, belongs to the Dicroglossidae family within the order Anura. Primarily found in southern China and northern Vietnam, it inhabits rocky streams in evergreen forests and open fields at altitudes ranging from 500 m to 1,500 m above sea level (Yu et al., 2016b). Due to its significant medicinal and nutritional value, there is a growing demand for its meat, which has driven the expansion of the frog farming industry in China (Shu, 2000). However, high-density farming practices have led to outbreaks of infectious diseases, including “rotting skin” disease (Liu et al., 2024), cataract disease, ascites disease, and meningoseptica bacteremia (Lei et al., 2019), caused by bacteria, viruses, and parasites. These diseases result in substantial economic losses and hinder the development of the *Q. spinosa* farming industry. Despite the critical role of *TLRs* in innate immunity, the composition and immune functions of the *TLR* family in *Q. spinosa* have yet to be characterized. Given the importance of *TLRs* in pathogen recognition, it is essential to explore the *TLR* family in this species further.

In this study, 17 *TLRs* were identified from the *Q. spinosa* genome database, and bioinformatic analyses were performed to investigate their gene structures and phylogenetic relationships. Additionally, *TLR* gene expression was analyzed at various time points following pathogen challenges in spleen tissue. The results provide essential genomic data to understand the potential functions of the *TLR* gene family in *Q. spinosa* and offer preliminary insights into the evolutionary mechanisms of *TLRs* in amphibian innate immunity.

## 2 Materials and methods

### 2.1 Identification of members of the TLR gene family

The complete whole genome data of *Q. spinosa* was downloaded from the DRYAD data platform (https://datadryad.org/stash) in order to identify members of the *TLR* gene family of species *Q*. *spinosa* (Hu et al., 2022). In addition, homologous *TLR* protein sequences of *H*. *sapiens*, *X*. *laevis*, *Nanorana parkeri*, *Rana temporaria* and *Danio rerio* were downloaded from the National Center for Biotechnology Information (NCBI) databases. They were used as query sequences for searching against the whole genome of *Q. spinosa* to identify candidate *TLR* family members via the TBLASTN of local Blast2.2-26 (Camacho et al., 2009), with an e-value of 1 × 10^−5^. The sequences of candidate *TLR* family members of *Q. spinosa* (*QsTLRs*) were obtained and further confirmed by performing a comparison between them and the NCBI protein sequence database.

### 2.2 Gene structure characterisation and protein-conserved domain prediction

The Expasy ProtParam tool was used to calculate the amino acid sequences of *QsTLRs* (Wilkins et al., 1999). The subcellular localisation prediction was performed using the WOLF PSORTY (Horton et al., 2007). Exon-intron structure of *QsTLRs* were analyzed using the online gene structure visualisation server GSDS (Hu et al., 2015). Conservative motifs were evaluated using MEME suite 5.5.5 online tools (Bailey et al., 2015) and the final genetic structures were visualised using TBtools local visual software. Protein conserved domains were identified and annotated using the normal mode of the online Simple Modular Architecture Research Tool (SMART) (Schultz et al., 1998) with all parameters at default levels. The TIR domains were compared using GeneDoc multi-sequence alignment software.

### 2.3 Phylogenetic and syntenic analysis of the TLR gene family in Q. spinosa

Molecular phylogenetic analysis was constructed based on the predicted amino acid sequences of *TLR* genes in *Q. spinosa* and the orthologous sequences in other representative vertebrates, which included *Anolis carolinensis*, *Chrysemys picta*, *D*. *rerio*, *Lateolabrax maculatus*, *H*. *sapiens*, *M*. *musculus*, *X*. *laevis* and *N*. *parkeri* (the corresponding *TLR* sequences that were used to create phylogenetic tree can be seen in Supplementary Table S1). Multiple sequences were aligned using the MUSCLE program in MEGA 11 with default parameters (Tamura et al., 2021). The tree was constructed using the neighbour-joining (NJ) method with a bootstrapping value setting of 1,000 replications (Saitou and Nei, 1987).

### 2.4 Analyses of conserved synteny

The reported genomic data of representative Anurans of *X*. *laevis* (ID: GCF_017654675) and *R*. *temporaria* (ID: GCF_905171775) were downloaded from the NCBI database by MCScanX (Wang et al., 2012) for homozygosity analysis in order to further investigate the conservation of the *QsTLR* genes in Anurans. The *TLRs* were also further conserved analysis with protein sequences of 50 KP neighbouring genes upstream and downstream of them and finally visualised using IBS2.0 software (Xie et al., 2022).

### 2.5 Challenge experiment and sample collection

The bacteria *Elizabethkingia miricola* is a common pathogen found in *Q. spinosa*, which causes the cataract disease and serious death of the frog. Here we selected the bacteria for conducting the challenge experiment (*E. miricola* was isolated from diseased *Q. spinosa* and stored in the laboratory) to characterize the immune responses of the *TLR* genes of *Q. spinosa* against bacterial infection.

*Q. spinosa* (average weight of 100 ± 10 g) were cultivated in a raising farm in Qingyuan City, Guangdong Province. Prior to the experiments, the frogs were acclimatised in circulating water with a temperature of 25°C for 2°weeks and fed daily with a common diet of yellow mealworm. The frogs were then divided into treated and PBS control groups, with 60 frogs in each group (54 frogs were used as experimental and the rest were supplemental). The treated group were all injected intraperitoneally with 0.1 mL (4.0 × 10^6^ CFU/mL) of *E. miricola* and frogs in the control group were injected with an equal amount of sterile PBS. To reduce the impact of random errors, improve the reliability and statistical validity of the experimental results, after infection, 5 frogs (N = 5) were chosen at random from both the control and treated groups after 0, 6, 9, 12 and 24 h and euthanised after being anaesthetized with MS-222 (Sigma, United States) before tissue dissection. Spleen, kidney, and liver tissues were sampled and immediately frozen in liquid nitrogen and stored in −80°C refrigerator for subsequent RNA extraction. All animal experiments were conducted in accordance with the guidelines for the care and use of laboratory animals that have been approved by the Institutional Animal Care and Use Committee in Zhongkai University of Agriculture and Engineering, Guangdong, China.

### 2.6 RNA extraction, cDNA synthesis and qPCR analysis

The total RNA was extracted from the spleen, kidney, and liver tissue using TRIzol reagent (Invitrogen, United States) and digested with RNase free DNase I (Thermo Scientific, United States) according to the instructions of the manufacturer. The quality of extracted RNA was examined by 1% agarose gel electrophoresis and NanoDrop One microvolume-uv-spectrophotometer (Thermo Fisher Scientific, United States) with an A260/280 ratio of between 1.8 and 2.1. The first strand cDNA was synthesised using HiScript II-RT SuperMix for qPCR (+gDNA wiper) (Vazyme, Nanjing, China) following the protocol and the cDNA products were stored at −20°C for further experiments.

Quantitative real-time PCR (qRT-PCR) was used for detecting the mRNA level of *TLR* genes using a CFX Connect Real-Time PCR Detection Systems (Bio-Rad, California, United States). The gene-specific primers for qRT-PCR were designed based on each *TLR* gene sequence using primer 5 software (Supplementary Table S2). The total volume of the qRT-PCR reaction was 20 μL, which included 10 µL of ChamQ SYBR qPCR Master Mix (2×), 1 µL of cDNA (3 times dilution of template), 0.4 µL of each primer (10 µM) and 8.2 µL of RNase-free water. The qRT-PCR of each sample was performed in triplicate according to the following conditions: denaturation at 95°C for 30 s, followed by 40 cycles of 95°C for 5 s and 60°C for 30 s. Melting curve analyses were performed at the end of each amplification to check the specificity of the reaction. The *β-actin* gene was amplified in parallel for normalisation.

### 2.7 Statistical analysis

All data were first subjected to Shapiro-Wilk normality test and Levene’s chi-square test. The number of threshold cycles (CT values) was collected and the 2-^△△CT^ method was used to calculate the expression levels of each gene. Significant differences between samples were assessed using one-way ANOVA followed by Tukey’s HSD *post hoc* correction. Expression patterns of differentially expressed genes were visualized using the R package pheatmap (version 1.2.12) (FDR-adjusted). After Z-score normalization, clustered heat maps were constructed using Euclidean distance and Ward.D2 clustering algorithm to elucidate the underlying patterns of gene expression.

## 3 Results

### 3.1 Identification and characterisation of QsTLRs in Q. spinosa

Following local identification and bioinformatic analysis, 17 different *TLR* genes were identified from the *Q. spinosa* genome database (Table 1): *QsTLR1*, *QsTLR2*, *QsTLR3*, *QsTLR4*, *QsTLR5*, *QsTLR5L*, *QsTLR6*, *QsTLR7*, *QsTLR8*, *QsTLR13a*, *QsTLR13b*, *QsTLR14a*, *QsTLR14b*, *QsTLR19a*, *QsTLR19b*, *QsTLR21*, *QsTLR22*. 17 *QsTLR* genes were located on 9 of the 13 largest scaffolds in *Q. spinosa* genome. The open reading frame (ORF) length of the 17 genes ranged from 1944 to 3138 bp, encoding 720 to 1046 amino acids. The physicochemical property analysis found the predicted molecular weights of *QsTLRs* to range from 71.29 to 120.56 kDa, and the theoretical pI values were between 5.66 and 8.81. Aliphatic analysis revealed most of the *QsTLR* proteins to be hydrophobic proteins, excluding *QsTLR7*, *QsTLR13a* and *QsTLR19a*. The predicted subcellular location suggested that most *QsTLR* proteins were targeted to the plasma membrane, with the exception of *QsTLR5L* which was extracellular proteins.

**TABLE 1:** Summary of TLR genes identified in *Q. spinosa*.

Name	Location	ORF (bp)	Amino acids	MW (kDa)	Theoretical pI	Aliphatic index	Subcellular location
*TLR1*	scaffold_01	2,440	813	94.81	6.95	103.68	Plasma membrane
*TLR2*	scaffold_01	2,361	786	89.96	5.81	106.59	Plasma membrane
*TLR3*	scaffold_01	2,694	897	102.54	7.05	104.46	Plasma membrane
*TLR4*	scaffold_08	2,526	841	96.12	6.94	104.98	Plasma membrane
*TLR5*	scaffold_04	2,388	795	92.37	5.91	103.43	Plasma membrane
*TLR5L*	scaffold_09	1944	647	71.29	6.52	105.15	extracellular
*TLR6*	scaffold_01	2,349	782	90.34	5.66	107.05	Plasma membrane
*TLR7*	scaffold_02	3,138	1045	120.56	8.34	99.44	Plasma membrane
*TLR8*	scaffold_02	3,141	1046	120.46	7.73	107.98	Plasma membrane
*TLR13a*	scaffold_11	2,163	720	84.37	7.31	98.54	Plasma membrane
*TLR13b*	scaffold_11	2,850	949	110.53	8.81	105.26	Plasma membrane
*TLR14a*	scaffold_13	2,526	841	96.66	5.37	102.21	Plasma membrane
*TLR14b*	scaffold_13	2,532	843	97.17	6.06	101.17	Plasma membrane
*TLR19a*	scaffold_03	2,841	946	110.27	5.8	98.37	Plasma membrane
*TLR19b*	scaffold_03	2,817	938	109.78	6.45	103.09	Plasma membrane
*TLR21*	scaffold_11	2,847	948	109.92	8.27	108.34	Plasma membrane
*TLR22*	scaffold_03	2,817	938	107.06	6.3	108.37	Plasma membrane

### 3.2 Phylogenetic relation of the TLR gene family among several vertebrates

**FIGURE 1:**
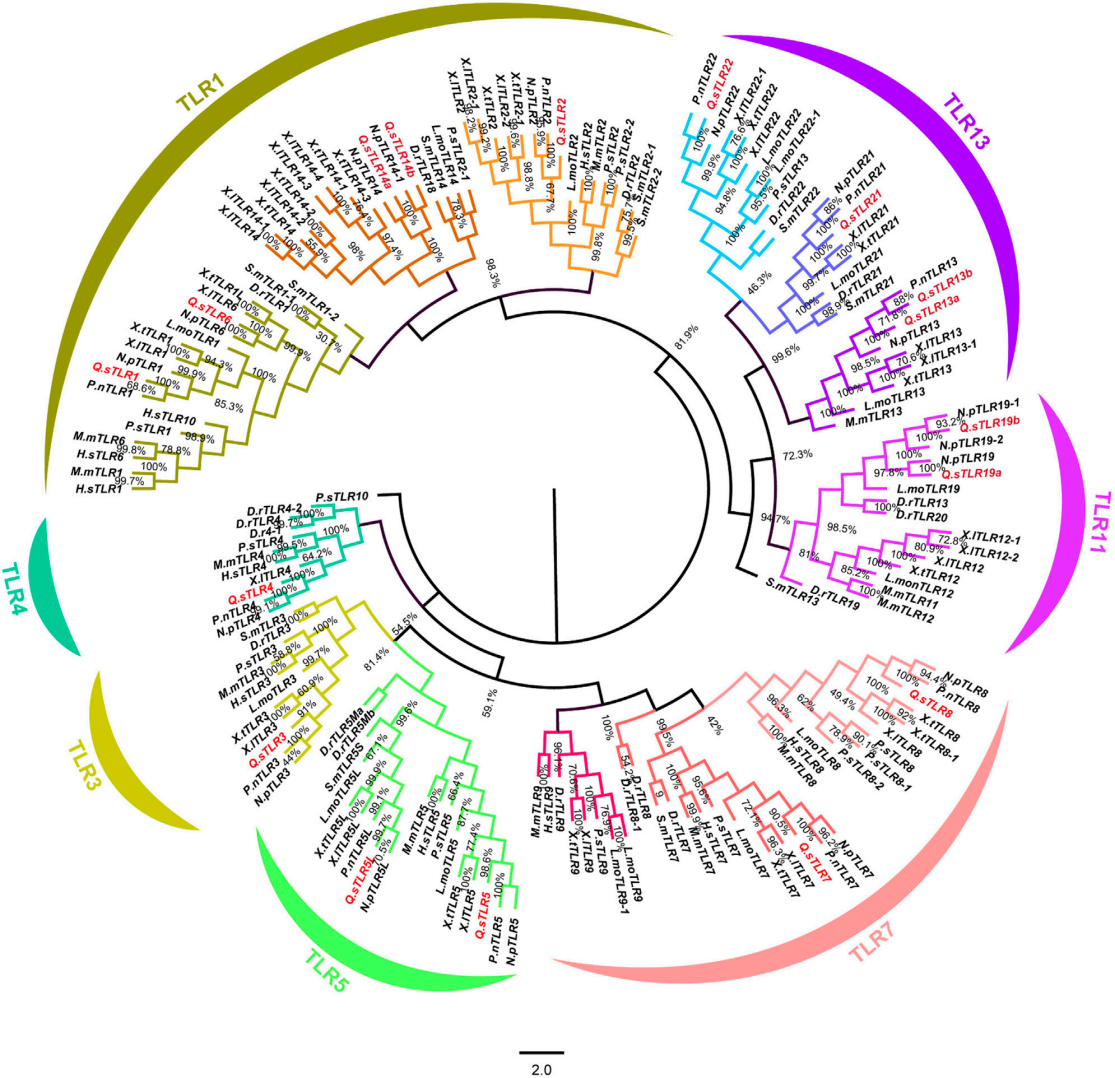
Phylogenetic tree of *TLR* gene families of 18 selected representative species. *TLR* genes of *Q. spinosa* were marked in red. The phylogenetic tree was divided into seven different subfamilies and each subfamily was marked in different colour.

A neighbour-joining (NJ) phylogenetic tree was constructed based on full-length amino acid sequences of *QsTLRs* and 18 other vertebrates to investigate the phylogenetic relationships of *TLRs* between *Q. spinosa* and other vertebrates (Figure 1). The phylogenetic tree revealed that all vertebrate *TLRs* were mainly clustered into seven major subfamilies and named *TLR1*-subfamily (*TLR1*/*1L*/*2*/*6*/*14a*/*14b*), *TLR3*-subfamily, *TLR4*-subfamily, *TLR5*-subfamily (*5*/*5M*/*5L*), *TLR7*-subfamily (*TLR7*/*8*/*9*), *TLR11*-subfamily (*TLR12*/*19*) and *TLR13*-subfamily (*TLR13*/*21*/*22*). The *TLR1* subfamily contained the maximum number of *TLR* members, including *TLR1*, *TLR1L*, *TLR2*, *TLR6*, *TLR14a* and *TLR14b*. The *TLR3* and *TLR4* subfamilies both contained only 1 *TLR* member, while the *TLR5* subfamily contained *TLR5*, *TLR5M*, *TLR5S* and *TLR5L* members. *Q. spinosa* had several representative *TLR* genes from major vertebrates and homologues to other vertebrate *TLRs* that were found to be highly conserved and clustered in the same subfamilies. However, no homologues of *TLR9* and *TLR12* were found in *Q. spinosa* genome in comparison to the *TLRs* of *Xenopus tropicalis* (Ishii et al., 2007). *TLR4*, which was missing in some fish such as *Siniperca chuatsi* (Wang et al., 2021), *L*. *maculatus* (Fan et al., 2019) and *Lethenteron japonicum* (Kasamatsu et al., 2010), in addition to the predicted *TLR4* of *X*. *tropicalis* (Ishii et al., 2007), was identified in *Q. spinosa*. The results show that *QsTLRs* clustered in the same branch as homologues of other vertebrate *TLRs*. High support rates among the seven *TLR* subfamilies suggest that they were both evolutionarily and functionally related to each other.

### 3.3 Analyses of conserved synteny

**FIGURE 2:**
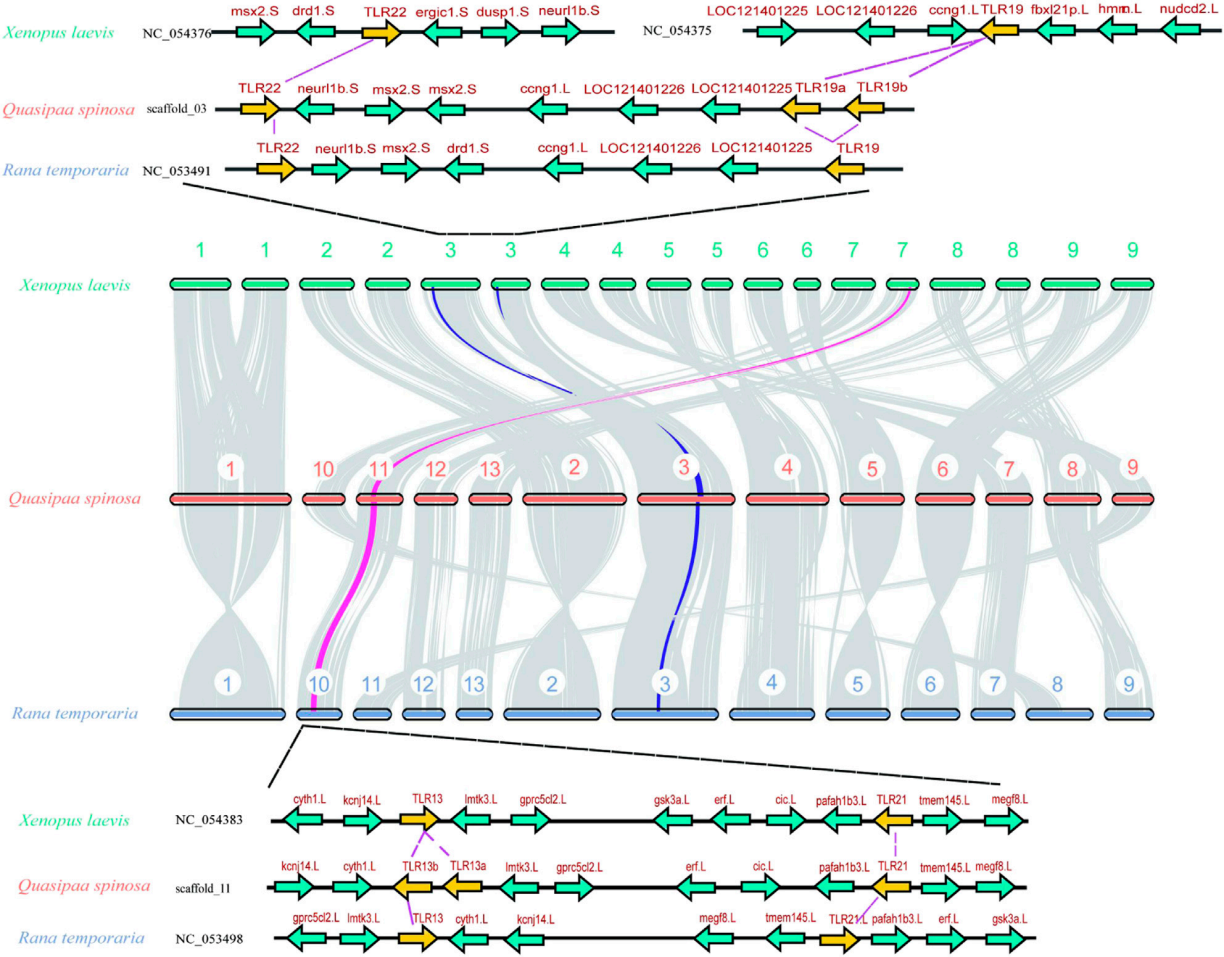
A Syntenic analysis of the chromosomes between *Q. spinosa*, *R*. *temporaria* and *X*. *laevis*. The conserved commonality manifested by some gene loci near representative *TLR* members.

Chromosomal homology analysis was conducted using *Xenopus laevis* and *Rana temporaria* as representative species (Figure 2) to investigate the homology of *TLR* genes in *Q. spinosa* within the Anuran order. The results revealed high chromosomal homology among the three species, with *Q. spinosa* showing chromosomal breakage and fusion events compared to *X. laevis*. Notably, a closer genetic relationship was observed between *Q. spinosa* and *R*. *temporaria*. To explore the conservation of the *TLR* gene family in these species, upstream and downstream genes of the *TLR* loci were analyzed (Figure 2; Supplementary Figure S1). The positions of *TLR* genes in Anurans appeared to be conserved, as the surrounding genes exhibited similar arrangements. However, some differences in the copy number and location of *TLR*s were observed across family members. For example, in *X*. *laevis*, *TLR*22 and *TLR*19 were located on different chromosomes, whereas in both *Q. spinosa* and *R*. *temporaria*, these two *TLRs* were located on the same chromosome, likely due to genetic variation and adaptation to different habitats.

### 3.4 Gene structure characterisation and protein domain prediction

**FIGURE 3:**
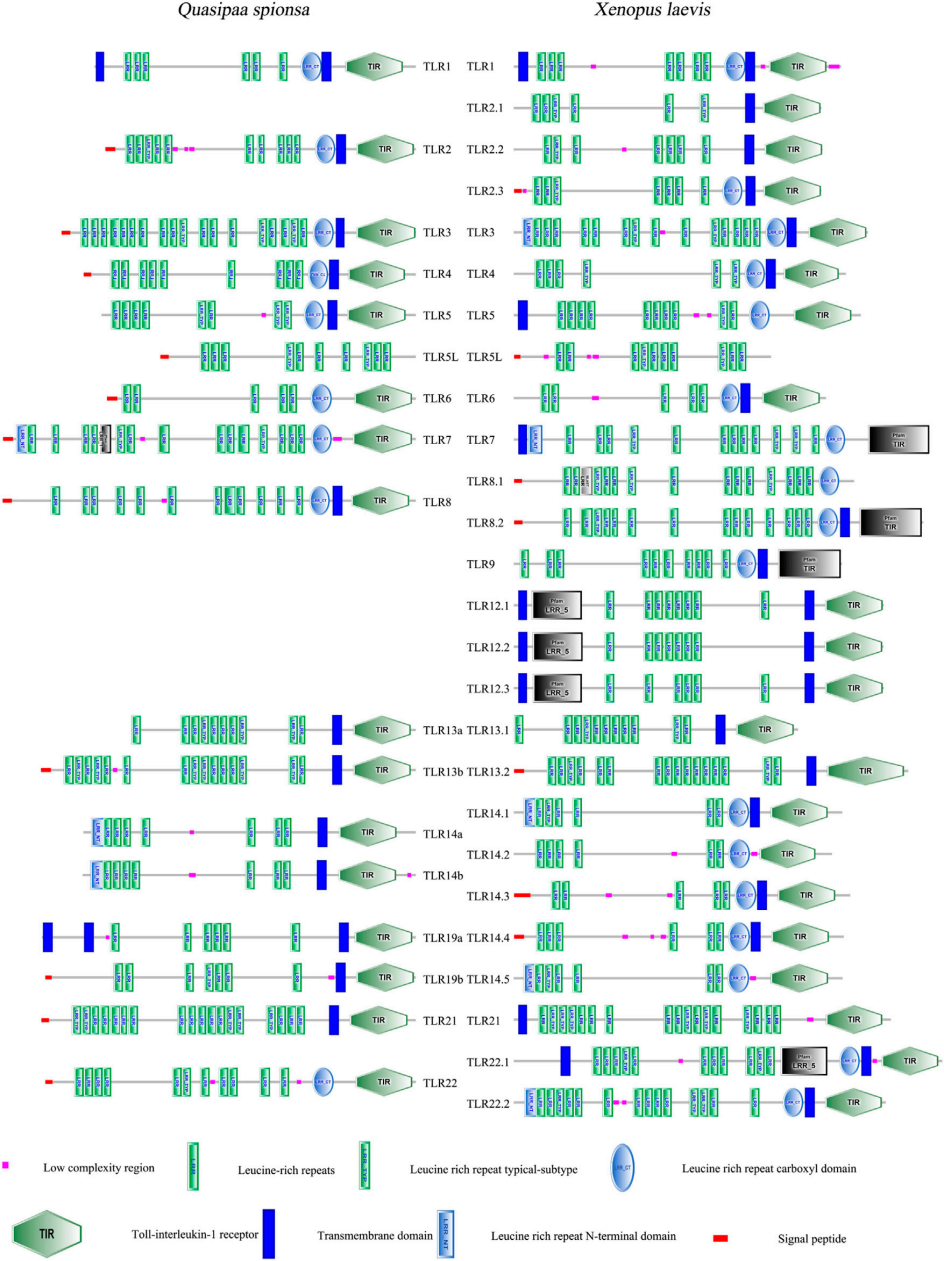
The structural feature prediction of *TLR* genes in *Q. spinosa*.

To further investigate the structural features of *QsTLRs*, protein models were predicted based on known *TLR* sequences from *X. laevis* using SMART software. The results (Figure 3) indicated that the proteins encoded by *QsTLRs* primarily consist of three functional domains: the LRR domain, the TM domain, and the intracellular Toll/interleukin-1 receptor (TIR) domain. All *QsTLRs* contained these three domains, with exceptions such as *QsTLR5L*, which lacked both the TM and TIR domains, and *QsTLR6*, *QsTLR7*, and *QsTLR22*, which lacked the TM domain.

The *TLR* proteins of *Q. spinosa* exhibited varying numbers of LRR domains, ranging from 5 to 18. This variation in LRR count may be linked to their distinct mechanisms for recognizing PAMPs. The intracellular TIR domain was identified as a crucial functional region for signal transduction (Oda and Kitano, 2006; Kawai and Akira, 2010). Comparative analysis of the TIR domains of *QsTLRs* (excluding *QsTLR5L*) revealed three highly conserved regions, named Supplementary Boxes 1–3. These conserved motifs suggest that the signal transduction mechanism is preserved across these *TLRs*, as highlighted in Supplementary Figure S2.

**FIGURE 4:**
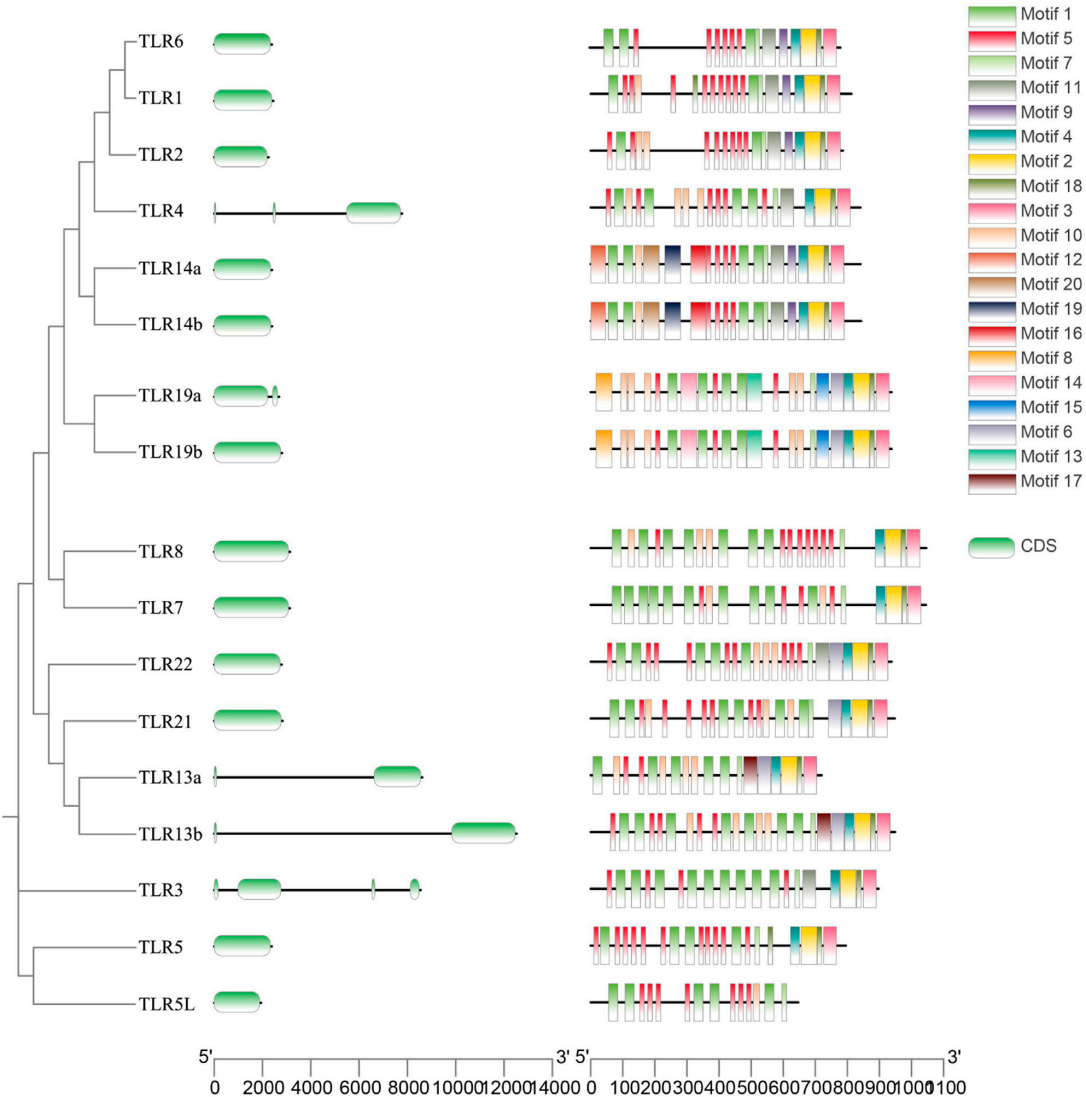
Gene structure characterization and conserved motifs of *TLR* genes in *Q. spinosa*.

A comparative analysis of the 17 *QsTLRs* identified 20 conserved motifs, which were organized according to their frequency of occurrence (Figure 4). Motifs 1, 5, and 7 were widely distributed across the *TLR* protein sequences of *Q. spinosa*. The distribution of motifs within the same subfamily, such as *QsTLR1*/*QsTLR6*, *QsTLR7*/*QsTLR8*, *QsTLR14a*/*QsTLR14b*, and *QsTLR19a*/*QsTLR19b*, was highly similar, suggesting functional conservation within subfamilies. Most *QsTLRs* contained a single exon, while *QsTLR13a*, *QsTLR13b*, and *QsTLR19a* had two exons, *QsTLR4* contained three exons, and *QsTLR3* had four.

### 3.5 Expression of TLR genes at mRNA level in Q. spinosa following the stimulation of E. miricola

**FIGURE 5:**
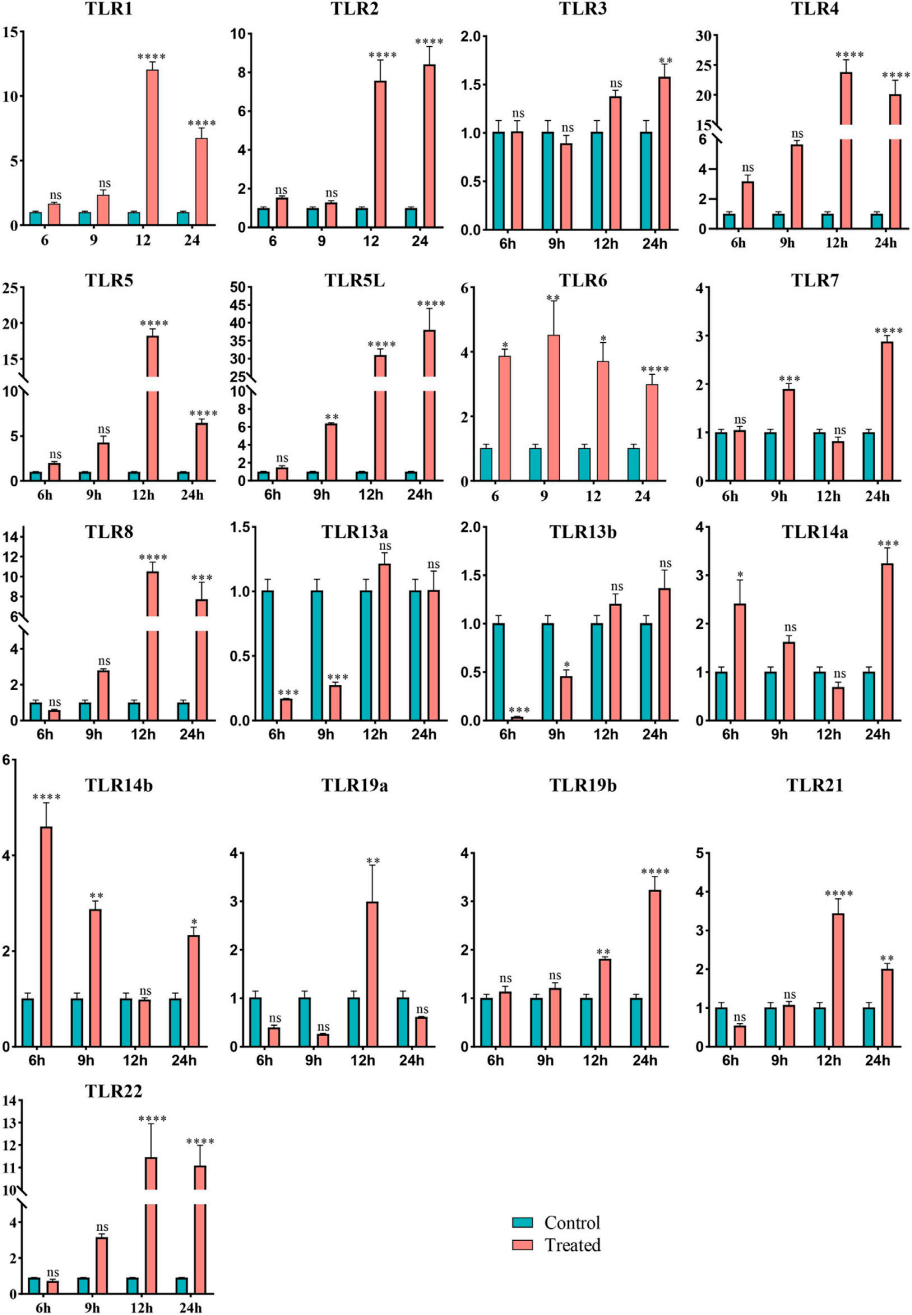
Expression profiles of *TLR* genes in *Q. spinosa* spleen tissue after *E. miricola* injection. Relative gene expression levels of *QsTLR*s were normalized to *β-actin*. Asterisks indicate statistically significant differences in upregulation/downregulation at different time points (*: *P* < 0.05, **: *P* < 0.01, ***: *P* < 0.001, ****: *P* < 0.0001).

To assess the potential function of *TLR* genes in *Q. spinosa* in response to bacterial infection, the expression profiles of 17 *TLR* genes were evaluated in spleen, kidney, and liver tissues following challenge with *E*. *miricola*. The results showed a significant upregulation of 15 *TLR* genes in spleen tissue at varying time points. However, no consistent patterns were observed in liver and kidney tissues (Supplementary Figure S3), likely because these tissues are not the primary immune organs in *Q. spinosa*. Consequently, the analysis focused on spleen tissue expression (Figure 5). *QsTLR1*, *QsTLR4*, *QsTLR8*, *QsTLR21*, and *QsTLR22* were significantly upregulated at 12 h (*P* < 0.001) and subsequently downregulated at 24 h (*P* < 0.001). *QsTLR6* was upregulated at 6 h (*P* < 0.05), while *QsTLR7* showed upregulation at 9 h (*P* < 0.001), both briefly downregulated at 12 h and then significantly upregulated again at 24 h. *QsTLR*3 exhibited continuous upregulation at all time points tested. For *TLR* genes with two subtypes, such as *QsTLR5*/*QsTLR5L*, *QsTLR13a*/*QsTLR13b*, and *QsTLR14a*/*QsTLR14b*, similar expression patterns were observed. *QsTLR5* and *QsTLR5L* were both significantly upregulated at 12 h (*P* < 0.01), with *QsTLR5* downregulated at 24 h (*P* < 0.001), while *QsTLR5L* remained continuously upregulated at 24 h. *QsTLR13a* and *QsTLR13b* were both significantly downregulated at 6 h (*P* < 0.001) and then gradually upregulated at subsequent time points. *QsTLR14a* and *QsTLR14b* were significantly upregulated at 6 h (*P* < 0.05), followed by a slow downregulation and then upregulation at 24 h. Expression patterns for *QsTLR19a* and *QsTLR19b* differed: *QsTLR19a* was downregulated after infection but significantly upregulated at 12 h, while *Qs*TLR*19b* showed continuous upregulation from 6 to 24 h.

Clustering analysis of the expression profiles categorized the *QsTLR* genes into four groups (Supplementary Figure S4). Type III, the largest group, included *QsTLR3*, *QsTLR7*, *QsTLR13a*, *QsTLR13b*, *QsTLR14a*, *QsTLR14b*, *QsTLR19a*, *QsTLR19b*, and *QsTLR21*. Type II consisted of *QsTLR1*, *QsTLR2*, *QsTLR6*, *QsTLR8*, and *QsTLR22*. *QsTLR4* and *QsTLR5* were classified as Type I, while *QsTLR5L* was separated as a distinct group, Type IV.

## 4 Discussion

*TLRs* have been identified in several vertebrates, including bovines (Fisher et al., 2011), birds (Velová et al., 2018), *D*. *rerio* (Chen et al., 2021), *Megalobrama amblycephala* (Lai et al., 2017), *N*. *parkeri* (Zhang L. et al., 2022), *X*. *tropicalis* (Ishii et al., 2007), *Pelophylax nigromaculatus* (Zhang L. et al., 2022) and *L*. *montandoni* (Babik et al., 2014). However, the nucleotide sequences of *TLRs* from *Q. spinosa* are currently unavailable in the NCBI database. In this study, 17 *QsTLR* sequences were identified from the whole genome of *Q. spinosa*, and their nucleotide and protein sequences were analyzed. Additionally, the expression profile of these *TLRs* was examined through qRT-PCR following *E. miricola* infection, aiming to elucidate the role of *QsTLRs* in pathogen resistance.

### 4.1 Potential reasons for variation of TLRs in amphibians

*TLR4* is present across most vertebrates, from fish to mammals (Velová et al., 2018). In fish, *TLR4* has been observed in Cypriniformes species with varying numbers of subtypes, such as three subtypes (*TLR4ba*/*bb*/*al*) in *D*. *rerio* (Chen et al., 2021), three subtypes (*TLR4*-*1*/*2*/*3*) in Cyprinus carpio(Gong et al., 2017) and one subtype in *M*. *amblycephala* (Lai et al., 2017), However, *TLR4* is absent in many other bony fish, such as Perciformes (Martínez-López et al., 2023). In reptiles and mammals, a single copy of *TLR4* is present (Zhou et al., 2016). In amphibians, *TLR4* has been detected in the genomes of *X*. *laevis* and *X*. *tropicalis* (Fisher et al., 2023), as well as in *Bombina maxima* transcriptomic data (Zhao et al., 2014). Similarly, one *TLR4* was identified in the *Q. spinosa* genome in this study. In contrast, no *TLR4* was found in the Caudata *L*. *montandoni* genome, suggesting that *TLR4* may be exclusive to Anuran amphibians (Babik et al., 2014). *TLR5* is present in various vertebrates and replicates across multiple species. Two types of *TLR5* are found in bony fish: soluble *TLR5S* and membrane-embedded *TLR5M* (Oshiumi et al., 2003). In mammals, including humans and mice, only the membrane-embedded *TLR5M* has been identified. However, amphibians (Babik et al., 2014) and some reptiles (such as turtles and the anole lizard (Abdullayev et al., 2013)), possess a variant known as *TLR5L*, which shares a similar protein structure to *TLR5S*. In this study, *TLR5L* was also found in *Q. spinosa*. Phylogenetic analysis showed that *TLR5L* clustered with *TLR5S* as a sister group, suggesting that *TLR5S* and *TLR5L* likely resulted from a duplication event of *TLR5* following the divergence of fish and amphibians. It is speculated that *TLR5S* and *TLR5L* may be functionally homologous. Furthermore, *TLR5* appears to be expanded in aquatic animals, such as fish and amphibians, compared to terrestrial animals, indicating its significant role in mediating resistance to the complex aquatic environments that these organisms inhabit.

The *TLR11* subfamily is both unique and complex, comprising four *TLR* genes: *TLR11*, *TLR12*, *TLR19*, and *TLR20*. In vertebrates, the existence of this subfamily spans from fish to mammals, although not all members are present in every group (Zhang L. et al., 2022). For instance, reptiles such as the Chinese soft-shelled turtle (Liu et al., 2019) and anole lizard lack members of the *TLR11* subfamily (Abdullayev et al., 2013). In other vertebrates, *TLR11* has only been identified in mammals, such as *M*. *musculus*. *TLR12* has been found in both mammals and amphibians, while *TLR20* exists only in fish, with multiple copies identified in species like *D*. *rerio* (six copies of *TLR20*) (Pietretti et al., 2014; Lv et al., 2023). *TLR19* has been observed in both fish and amphibians but is absent in amniotes (Wang et al., 2015; Gong et al., 2017; Zhang L. et al., 2022). In *Q. spinosa*, *TLR11* and *TLR12* were not identified, but two copies of *TLR19* (*a* and *b*) were found within the *TLR11* subfamily. Phylogenetic analysis showed that the two *TLR19* genes clustered with *TLR19* genes from other species, and *TLR19* was closely related to *TLR12*, suggesting that these genes may share homologous roles in host defense against pathogenic microorganisms.

Amphibians, as ancient vertebrates, occupy a critical evolutionary position, acting as a “bridge” between aquatic and terrestrial vertebrates (Inger et al., 1986). Compared to other animals, amphibians exhibit increased copy numbers of certain *TLR* genes, and some *TLR* genes are unique to fish and amphibians, being absent in other terrestrial vertebrates. For instance, *TLR13* appears in multiple copies in both fish and amphibians (Ishii et al., 2007; Liu et al., 2019; Wang et al., 2021; Zhang L. et al., 2022), while only a single copy is retained in reptiles and mammals (Song et al., 2015; Yu et al., 2016a). Similarly, *TLR14* exists in one or two copies in amphibians (Zhang L. et al., 2022), but only one copy is found in most reptiles. Interestingly, *TLR14* genes are absent in fish, birds, and mammals. *TLR19*, previously thought to be unique to bony fish with one copy, has been shown to also exist and expand in amphibians (Wang et al., 2015; Altmann et al., 2016; Qi et al., 2017; Liu et al., 2019). Multiple copies of *TLR22* are found in fish, with species such as *Gymnocypris eckloni* having two copies (*TLR22a* and *TLR22b*) (Qi et al., 2017), *Boleophthalmus pectinrostris* having four copies (*TLR22a* to *d*) (Qiu et al., 2019) and *Coregonus maraena* possessing as many as ten copies (*TLR22a* to *j*) (Altmann et al., 2016 *)*. In amphibians, one or two copies of *TLR22* remain, but it is completely absent in birds and mammals (Babik et al., 2014). The expansion and diversity of *TLRs* in cephalochordates and echinoderms are believed to reflect the evolutionary response to a variety of microorganisms and pathogens in aquatic environments (Rast et al., 2006; Huang et al., 2008; Messier-Solek et al., 2010). Similarly, the diversity of *TLRs* in amphibians plays a pivotal role in their ability to resist pathogenic bacteria during life domain migration.

### 4.2 Characterization of TLRs in Q. spinosa

Similar to other vertebrates, the proteins encoded by *QsTLRs* possess three typical functional regions: the extracellular region, the TM region, and the C-terminal intracellular region (TIR) (Figure 3). The extracellular region consists of 2–25 LRR domains, each containing 20 to 30 amino acids. These LRRs are essential for recognizing PAMPs in pathogenic organisms, such as bacteria, parasites, and fungi (Uematsu and Akira, 2006). The variation in the number of LRRs among species and *TLR* family members appears to be an evolutionary adaptation, enabling hosts to better recognize and respond to diverse PAMPs. Consequently, there is considerable variability in the number of LRRs across different *TLRs*. The TIR domain, which plays a vital role in *TLR* signaling, is highly conserved in *Q. spinosa* (O'Neill and Bowie, 2007). The functional conservation of the TIR domain across *TLR* members is mainly concentrated in three critical motifs (Supplementary Figure S2): Supplementary Box 1 ((F/Y) DAFISY), Supplementary Box 2 (LC---RD---PG), and Supplementary Box 3 (a conserved (FW) surrounded by basic residues). Notably, phenylalanine (F) in Supplementary Box 1 can be substituted with tyrosine (Y). This study found that proteins encoded by *QsTLRs* retain the important motifs of the TIR structural domain. Interestingly, proline (P) in Supplementary Box 2—an essential residue with auxiliary recognition functions—was found to be conserved in various fish species, such as *yellowtail leucocytes* (Reyes-Becerril et al., 2016), *Sebastiscus marmoratus* (Zhang Y. et al., 2022), *Larimichthys crocea* (Sun et al., 2016) and *Seriola lalandi* (Reyes-Becerril et al., 2016). This suggests that variations in conserved residue sites between amphibians and fish are not coincidental.

### 4.3 Immune response of QsTLRs against E. miricola infection

Previous research has demonstrated the functional similarity of factors involved in resistance to viral or non-viral exogenous attacks and in signaling cascade transduction, despite being derived from different species (Liu et al., 2020). This functional similarity is particularly evident among fish, reptiles, and mammals. However, the role of the *TLR* family in amphibians remains unexplored, and the functional comparison of *TLR* genes between amphibians and other vertebrates is not yet understood.

*E*. *miricola* is a common pathogen in *Q. spinosa* as well as other frogs, including *P*. *nigromaculatus* (Hu et al., 2017; Li et al., 2023), *Lithobates pipiens* (Trimpert et al., 2021), and *Rana catesbeiana* (Wei et al., 2023). Infected *Q. spinosa* exhibits typical symptoms such as cataracts with white, cloudy eyes, and reduced movement (Lei et al., 2019). The spleen, kidney, and liver often become enlarged or hemorrhagic (Li et al., 2023; Wei et al., 2023). This disease outbreak leads to high mortality rates in frog farming. Therefore, investigating the roles of *QsTLRs* in the immune response following *E. miricola* infection could provide valuable insights into enhancing innate immunity and developing immune adjuvants. In this study, *E. miricola* was selected as the pathogen for challenge experiments, and the expression levels of 17 *TLR* genes were assessed in the spleen, kidney, and liver tissue, to explore the potential anti-pathogenic immune responses of *TLR*s in *Q. spinosa*.

The results revealed significant temporal variation in the expression of most genes, suggesting their involvement in the immune response to *E. miricola* infection. *TLR2* and *TLR4* in fish and reptiles have been shown to recognize and respond to Gram-negative bacterial invasions, such as *Aeromonas hydrophila*, with activation occurring in the spleen (Zhang et al., 2013; Lai et al., 2017; Samanta et al., 2017; Liu et al., 2019). In this study, the expressions of *QsTLR2* and *QsTLR4* followed an upregulation trend, peaking at 12 h post-infection, similar to the expression patterns observed in Chinese soft-shelled turtle after *A. hydrophila* infection (Liu et al., 2019), indicating their active participation in the immune response to *E. miricola* in *Q. spinosa*. *TLR5* in fish and reptiles, such as *Anolis carolinensis*, is known to recognize bacterial flagellin and LPS, initiating immune responses in the spleen (Voogdt et al., 2016; Wang et al., 2021; Zhang Y. et al., 2022). In *Q. spinosa*, both *QsTLR5* and *QsTLR5L* exhibited a significant and sustained upregulation until 12 h post-infection, indicating that *TLR5* functions similarly across vertebrates in immune responses triggered by bacterial pathogens. However, while *QsTLR5* expression decreased at 24 h, *QsTLR5L* remained upregulated, suggesting that the two *TLR5* genes in *Q. spinosa* play critical roles in the immune response to *E. miricola* infection, with *QsTLR5L* contributing functional diversity and complexity.

Genes undergoing whole-genome duplication (WGD) or local replication events typically avoid gene loss through subfunctionalization or neofunctionalization (Jaillon et al., 2004). Fish *TLR13* is upregulated in response to poly (I:C), LPS, and PNG stimulation (Wang et al., 2021), with a similar response detected in the spleen of *Pelodiscus sinensis* (Liu et al., 2019). In mammals, such as mice, *TLR13* is activated by bacterial 23S rRNA and viral ssRNA, suggesting that *TLR13* in mammals, reptiles, and fish may share functional similarities (Song et al., 2015). However, antibiotic-resistant bacterial 23S rRNA and synthetic oligonucleotides containing methylated adenosine or guanosine have been shown to inhibit proper activation of *TLR13* (Oldenburg et al., 2012). In this study, *QsTLR13a* and *QsTLR13b* were significantly downregulated following infection, returning to baseline levels at 12 h, a response distinct from that observed in fish or mammals. This may indicate that *E. miricola* employs immune evasion strategies that prevent normal immune recognition by *TLR13*. In fish, *TLR19* is known to recognize dsRNAs (e.g., poly (I:C)) (Zhang Y. et al., 2022) and bacteria stimuli (Zhang et al., 2013). In this study, *QsTLR19b* exhibited a continuous upregulation at 24 h after infection, similar to the expression patterns seen in *Ictalurus punctatus* (Zhang et al., 2013). However, *QsTLR19a* displayed a distinct expression pattern, with upregulation only observed at 12 h, suggesting that *TLR19a* may function in a temporally regulated manner or be subject to more complex regulatory mechanisms. Although subtypes resulting from gene replication display some functional divergence, these differences may reflect evolutionary adaptations in response to the challenges posed by complex habitats.

Gene clustering analysis was performed to investigate the expression patterns of different subfamily members. The results showed that *QsTLR5* and *QsTLR4* exhibited highly similar expression profiles, suggesting they may work synergistically to recognize different receptor molecules. This complementary function could enable the detection of multiple signaling pathways (like downstream signaling), thereby preventing immune escape. *QsTLR5L* also responded to *E. miricola* infection and triggered an immune response; however, *QsTLR5L* did not cluster with *QsTLR5* but instead formed a distinct branch with consistently upregulated expression. This indicates that *QsTLR5L* functions independently of *QsTLR5* and may represent a fish-like variant, possibly an evolutionarily adapted *TLR5S* in frogs, tailored to meet the immunological demands of their complex aquatic and terrestrial environments. Further studies on *TLR5S* in fish and *TLR5L* in amphibians are needed to confirm this hypothesis.

In terms of functional analysis, the expression of *QsTLR* genes following *E. miricola* stimulation was initially examined. However, due to the absence of viral pathogens in the study, immune responses in *QsTLRs* to viral stimulation could not be assessed. Additionally, the expression profiles of *QsTLRs* were only analyzed in spleen tissues post-infection, which constitutes a limitation of this study. Although the expression of *QsTLRs* in liver and kidney tissues was also analyzed, the results were inconsistent, lacking any clear trends. This suggests that the kidney and liver may not be primary immune tissues in frogs, failing to generate immune responses after infection. A similar observation was made in *P*. *sinensis*, where immune responses to *A. hydrophila* were only detected in the spleen, not in the liver or kidney (Zhou et al., 2016; Liu et al., 2019). Therefore, only the spleen expression results are presented here. Despite the lack of further functional characterization, this study provides the first expression profile of *Q. spinosa* following *E. miricola* infection and identifies key *TLR* members (*TLR4*, *TLR5*, and *TLR5L*) likely involved in the immune response. This lays the groundwork for future studies exploring the role of *TLRs* in pathogen recognition in other frog species and investigates the complementary roles of different *TLR* members in downstream signaling pathways.

In conclusion, 17 *QsTLRs* were identified in *Q. spinosa*, offering a preliminary understanding of the *TLR* gene family in this species. The expression changes of these genes in response to *E. miricola* infection were analyzed, providing a basis for further research into the role of *Q. spinosa* in defending against exogenous pathogens.

## 5 Conclusion

In this study, 17 members of the *TLR* gene family were identified from the whole genome sequences of *Q. spinosa*. Phylogenetic analysis showed that the *QsTLRs* were highly homologous with their homologs in other vertebrates. Analysis of protein structural domains and motifs showed that the *QsTLRs* proteins were highly structurally conserved. qRT-PCR results showed that 17 *QsTLRs* responded positively to the attack by *E. miricola*, with different regulatory tendencies. 15 *QsTLR* genes displayed upregulation trends at different time intervals. Overall, these results provided a comprehensive understanding of the *QsTLRs* and provided a theoretical basis for further investigation on the immunological function as well as vaccine development in Chinese spiny frog.

## Data Availability

The datasets presented in this study can be found in online repositories. The names of the repository/repositories and accession number(s) can be found in the article/Supplementary Material.
