# Supplementary material for: Genome-wide identification and characterisation of Toll-like receptors in Chinese spiny frog (Quasipaa spinosa)
Source: Front Genet. 2025 Jun 6;16:1569669. doi: 10.3389/fgene.2025.1569669 (PMC12179096; doi:10.3389/fgene.2025.1569669)
Supplement: Supplementary file 1 [file Supplementaryfile1.docx]

**Supplementary Information**

**Supplementary Tables**

**Table S1. Representative vertebrates species list for use in Phylogenetic tree**

(*See the separate Excel workbook*)

**Table S2** Primers used for real-time qRT-PCR amplification of *QsTLRs*.

| Gene name | Forward primer 5'-3' |  | Reverse primer 5'-3' |
| --- | --- | --- | --- |
| *β-actin* | GAGCAGGACTGGGTGICTTCA |  | GCATTGTAACCAACTGGGACG |
| *TLR1* | GGTGGCTGGATTTGTCTA |  | TCAATATGGAGCACGTTTA |
| *TLR2* | ATCTAACCCTATTGCCGAACA |  | TAAGTCGTGCTGCACTCTTG |
| *TLR3* | GATTCATCCAGGGTGTTT |  | ACTTCAATCTGAGTTACGG |
| *TLR4* | ATCGTCGCCATTATGTTTC |  | GCCTTACCCATTCCTCGT |
| *TLR5* | TAAATCTGAGGGAGAACAAG |  | AAGGATAACAGGTGGACAAT |
| *TLR5L* | TTGGAGCAATGGATGGTCTT |  | GCAGTTAGGGCAGAAGGAGG |
| *TLR6* | GCAGCCTTCAAGAAAGTGAT |  | GCGGAGGTTGTTGTATGAGA |
| *TLR7* | ATAACCCGACTGTTTCCTCC |  | TTTACAGCGATGTGCTTGAT |
| *TLR8* | ACCGTCTGGATGTATTATGGA |  | ACAATGCTTCTTCGGGTATG |
| *TLR13a* | ATCTTACCCTGGACAACAAC |  | GAAAGAGGATTCATAGAGGC |
| *TLR13b* | ATGCCATAAATAATAGTCGGA |  | TAGGCTGAAAGTCTGTAATCTG |
| *TLR14a* | TTCGTCGTCATTGTCTGC |  | CAATCAGCGTCCGAATAG |
| *TLR14b* | CCTCTACAAATGGGCTACT |  | TGTCCAGCACCAAGAAAG |
| *TLR19a* | CTAAAGCAGAAATGCCAAGTA |  | TGTCCACCGTGTCACAATAA |
| *TLR19b* | ATGGCTGGAATCTAACACA |  | CGTTCATAGAGGAGGGATA |
| *TLR21* | TGATGCGTTTGTTTCCTA |  | CTCCACGATGTTGTCTATGA |
| *TLR22* | ATCTTCCTCCAACAACATCC |  | AACTTCCCTTCTCAACGACT |

**Supplementary Figures**


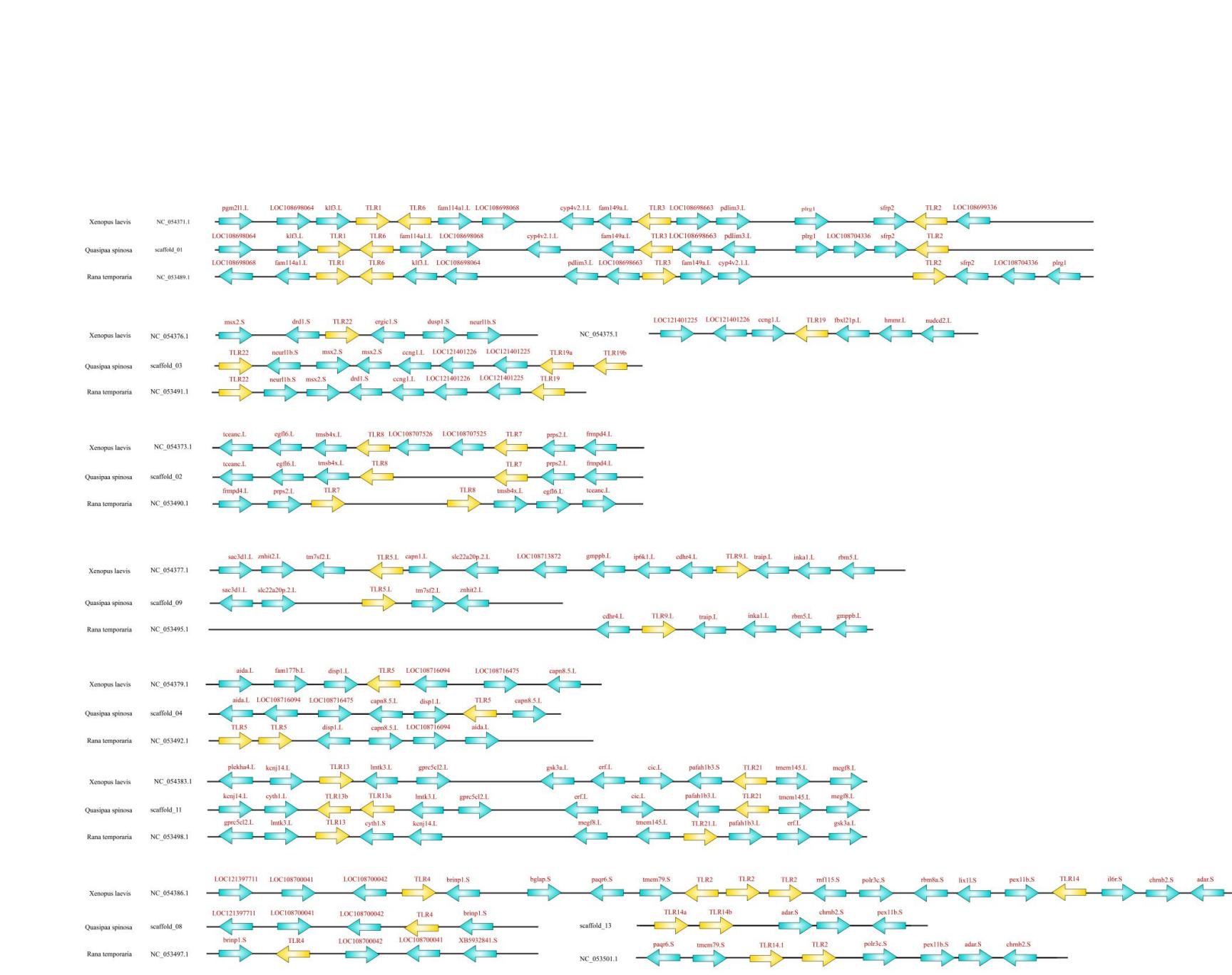


**Figure S1** Localisation of upstream and downstream neighbour genes of three amphibian *TLRs*.


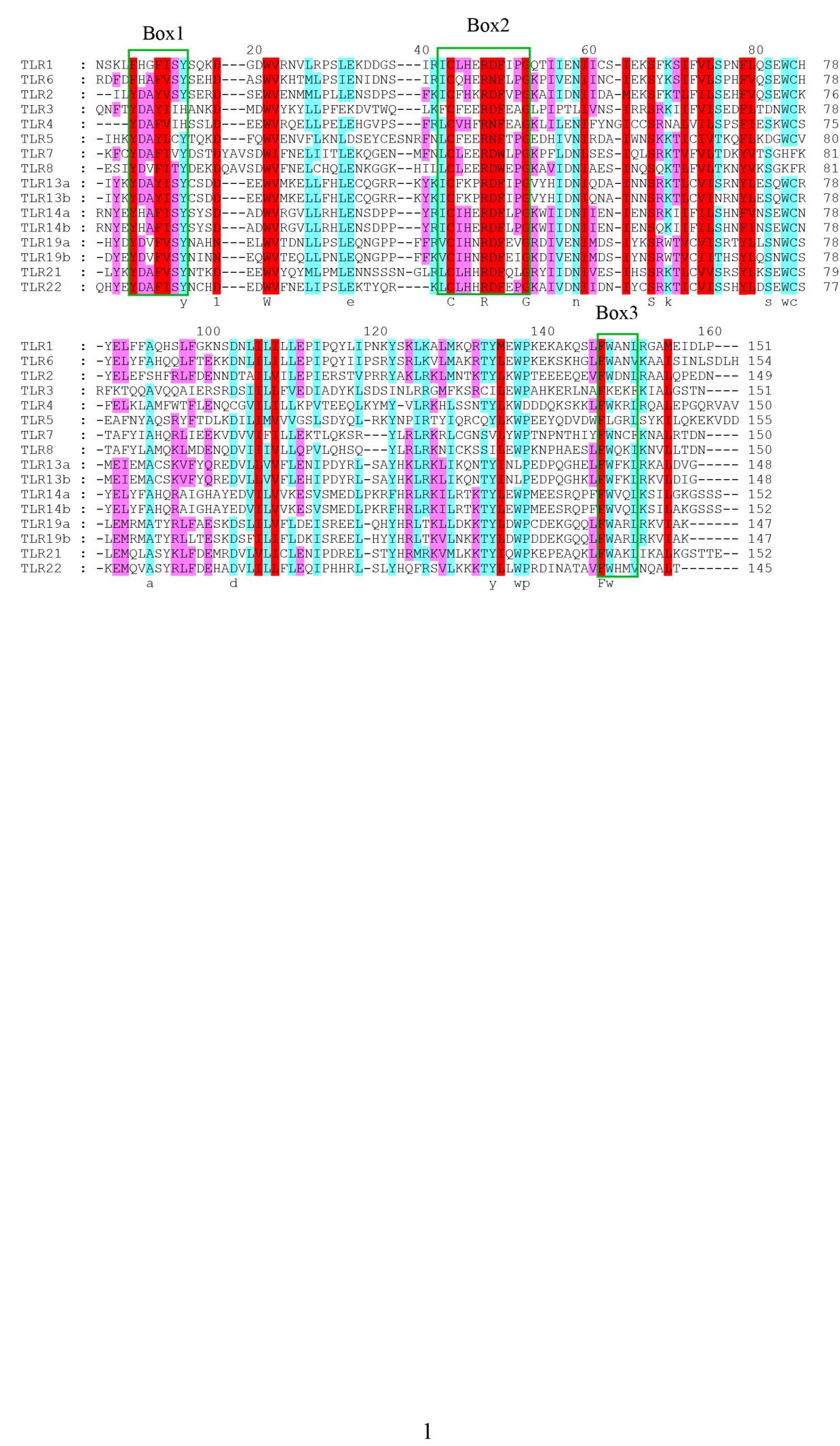


**Figure S2** Multiple sequence alignment of the TIR domain in *TLR* genes of *Q. spinosa*

*
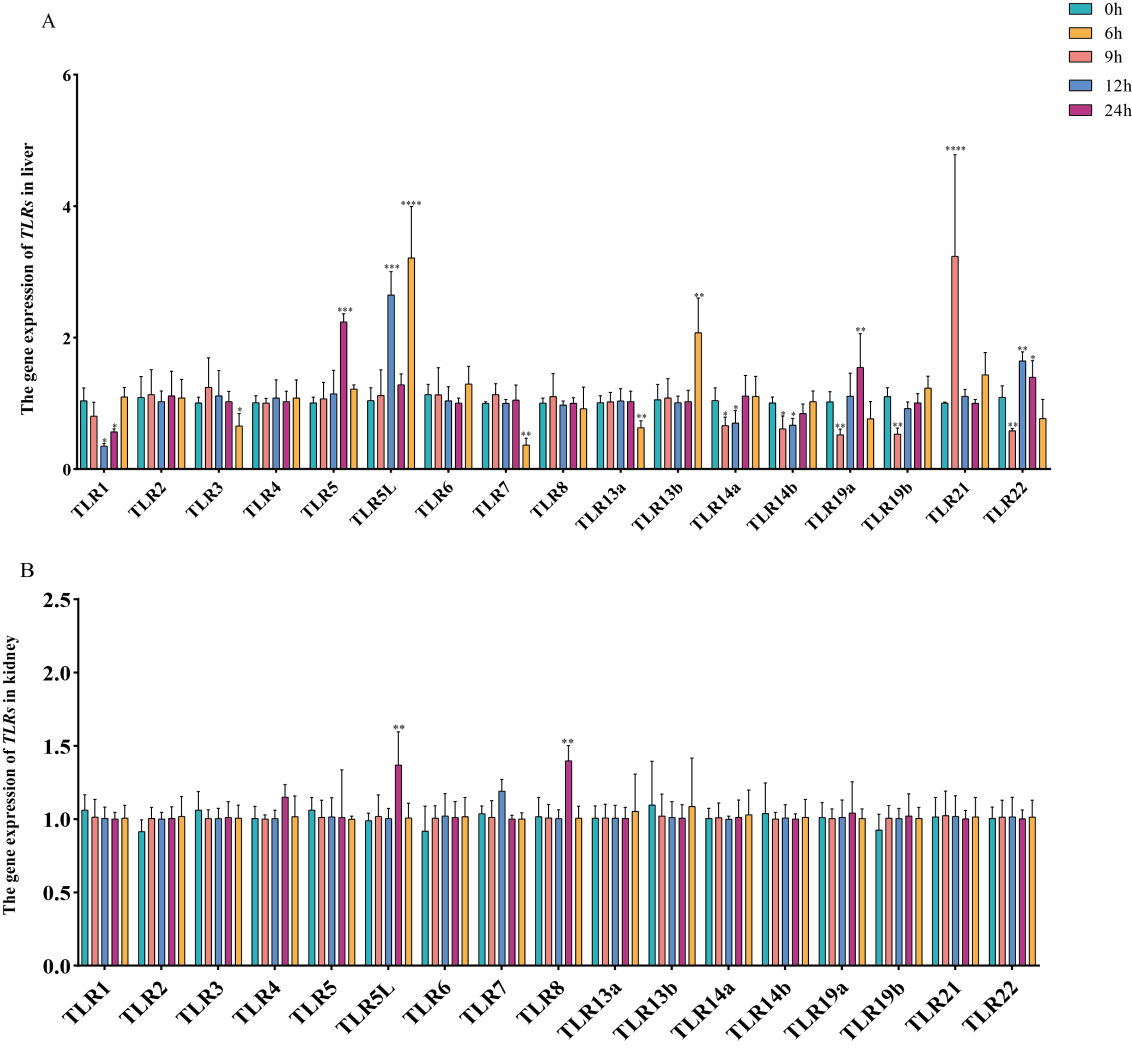
*

**Figure S3** Expression profiles of *TLR* genes in *Q. spinosa* liver and kidney tissues after *E. miricola* injection. Relative gene expression levels of *QsTLRs* were normalized to *β-actin*. Asterisks indicate statistically significant differences in upregulation/downregulation at different time points (*: *P* < 0.05, **: *P* < 0.01, ***: *P* < 0.001 and ****: *P* < 0.0001).


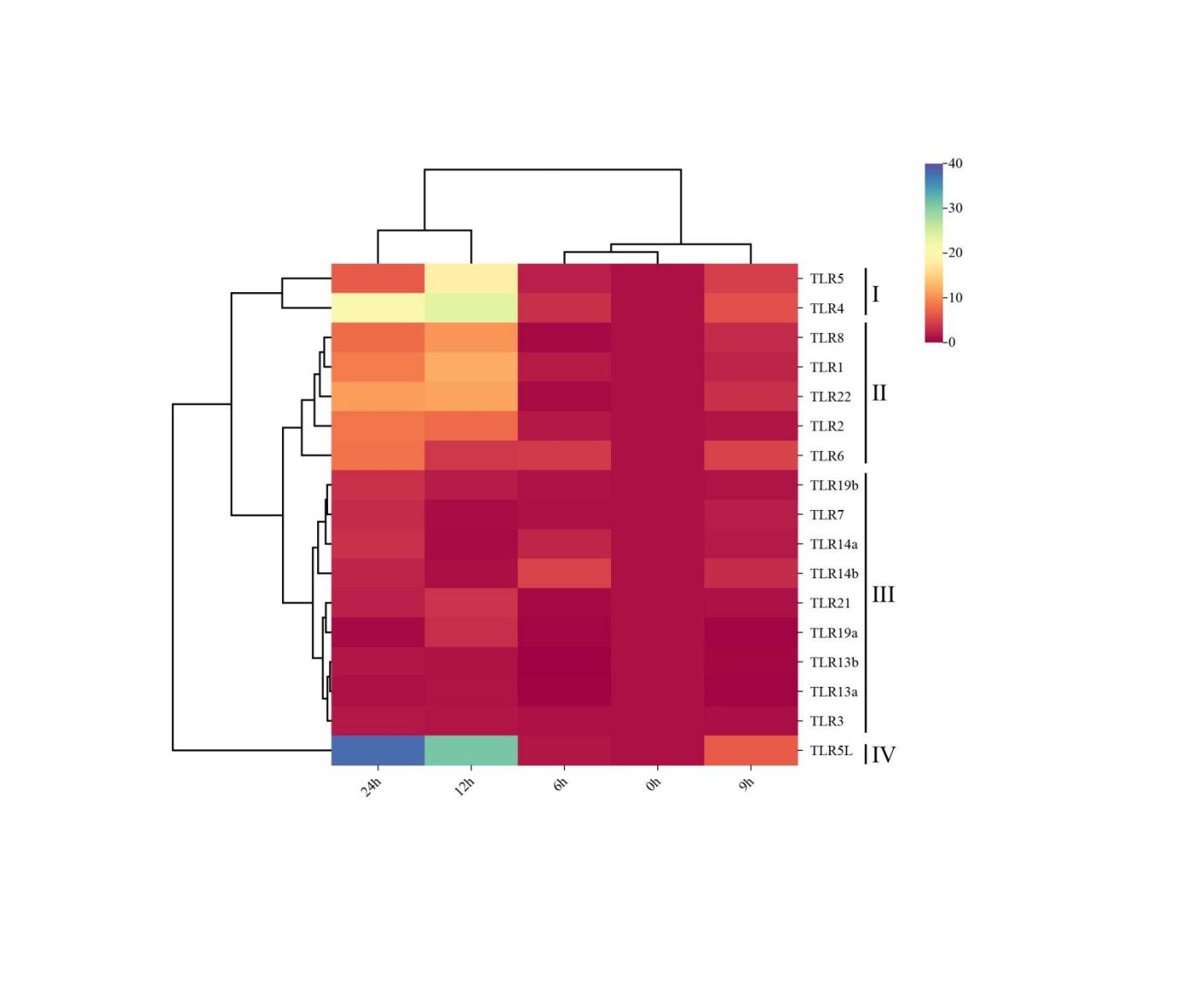


**Figure S4** A gene clustering analysis of *QsTLR* genes after *E. miricola* stimulation.
